# Factors associated with survival in adult patients with traumatic arrest: a retrospective cohort study from US trauma centers

**DOI:** 10.1186/s12873-021-00473-9

**Published:** 2021-07-05

**Authors:** Abdel-Badih Ariss, Rana Bachir, Mazen El Sayed

**Affiliations:** 1 Department of Emergency Medicine, American University of Beirut Medical Center, Riad El Solh, P.O. Box - 11-0236, Beirut, 1107 2020 Lebanon; 2 Emergency Medical Services and Prehospital Care Program, American University of Beirut Medical Center, Beirut, Lebanon

**Keywords:** Traumatic arrest, Survival, Outcome, Injury, Resuscitation

## Abstract

**Background:**

Traumatic arrests increasingly affect young adults worldwide with low reported survival rates. This study examines factors associated with survival (to hospital discharge) in traumatic arrests transported to US trauma centers.

**Methods:**

This retrospective cohort study used the US National Trauma Databank 2015 dataset and included patients who presented to trauma centers with “no signs of life”. Univariate and bivariate analyses were done. Factors associated with survival were identified using multivariate regression analyses.

**Results:**

The study included 5980 patients with traumatic arrests. Only 664 patients (11.1%) survived to hospital discharge. Patients were predominantly in age group 16–64 (84.6%), were mostly males (77.8%) and white (55.1%). Most were admitted to Level I (55.5%) or Level II trauma centers (31.6%). Injuries were mostly blunt (56.7%) or penetrating (39.3%). The median of the injury severity score (ISS) was 19 (interquartile range [IQR]: 9–30). Factors associated with decreased survival included: Age group ≥ 65 (Ref: 16–24), male gender, self-inflicted and other or undetermined types of injuries (Ref: assault), injuries to head and neck, injuries to torso and ISS ≥ 16 (Ref: < 16) and ED thoracotomy. While factors associated with increased survival included: All injury mechanisms (with the exception of motor vehicle transportation) (Ref: firearm), injuries to extremities or spine and back and all methods of coverage (Ref: self-pay).

**Conclusion:**

Patients with traumatic arrests have poor outcomes with only 11.1% surviving to hospital discharge. Factors associated with survival in traumatic arrests were identified. These findings are important for devising injury prevention strategies and help guide trauma management protocols to improve outcomes in traumatic arrests.

**Level of evidence:**

Level III.

## Background

Traumatic arrests (TA) or traumatic deaths are increasingly affecting the young population worldwide. According to a 2014 report by the World Health Organization (WHO), more than 5 million individuals die from injuries each year which accounts for approximately 9% of global deaths [1]. Main causes of fatal injuries are road traffic injuries, homicide and suicide [1]. TA has also increased in the US by 22.8% between the years 2000 and 2010, affecting mainly young adults [2]. As a result, traumatic fatalities compete with cancer and heart related mortality in the young population [2].

Prior studies that examined traumatic arrests have reported very low survival rates ranging from 1.5 to 12.5% [3–5]. Guidelines to withhold resuscitative measures specific for traumatic arrests have also been proposed in previous studies and by major organizations such as National Association of Emergency Medical Services Physicians and the American College of Surgeons Committee on Trauma (NAEMSP/ACS-COT) because of the futility of resuscitative measures for this condition [6, 7]. Survival has however been increasing with the improvement of medical interventions as well as the establishment of trauma systems [8, 9].

Previously identified factors that may contribute to increased survival in traumatic arrests include emergency department thoracotomy [9], presence of VF on admission [10, 11], witnessed TA [11], pre-hospital chest decompression [12], admission to trauma center [5, 13], lower injury severity score (ISS), high Glasgow Coma score, Caucasian race and higher systolic blood pressure (SBP) [5].

With evolving trauma care, improved survival is expected and factors associated with survival might change. This study used the 2015 dataset from the US National Trauma Databank (1) to examine characteristics and outcomes of patients suffering from traumatic arrests and (2) to identify factors associated with survival in traumatic arrests victims who were treated in US trauma centers.

## Methods

### Study design

This retrospective cohort study used the 2015 public release dataset of the National Trauma Data Bank (collected in 2015 and released in 2017).

An Institutional Review Board Exemption was obtained at the American University Of Beirut to use this de-identified dataset.

### Study setting

The National Trauma Database represents the largest U.S trauma data registry, is maintained by The American College of Surgeons Committee on Trauma and collects information from more than 900 facilities across the U.S. This registry includes patients who sustained one or more injuries that resulted in death, transfer or admission to a hospital and who have a trauma related diagnosis code (ICD-9 CM (800-959.9) or ICD-10 CM (S00-S99, T07, T14, T20-T28, T30-T32 and T79.A1-T79.A9) except cases with ICD-9CM codes 905-909.9, 910-924.9 and 930-939.9 and ICD-10CM codes (S00, S10, S20, S30, S40, S50, S60, S70, S80, S90) [14].

The variables included in the database include patient related information (demographic, coverage, outcome), event related information (injury severity, characteristics, diagnosis, pre-hospital, ED and hospital information) in addition to other information related to quality assurance and process of care measures. The injury severity score was categorized into two groups (≤ 15, ≥ 16) to classify all patients as having minor or major trauma [15].

### Study population

The 2015 NTDB data set contains 917,865 patients of which 8026 presented to the ED with “no signs of life” defined as “having none of the following: organized EKG activity, pupillary responses, spontaneous respiratory attempts or movement, and unassisted blood pressure” [16]. These patients were assumed to have CPR in progress on admission to the ED as per the NTDB dictionary and were considered to have sustained a traumatic arrest. Only adult patients were included and they were defined similarly to previous studies as those aged 16 years and above [17]. Patients were excluded if age was not recorded (n = 443), if they were transferred from other hospitals (recorded as inter-facility transfer; *n* = 521) and if outcome was unknown (ED discharge disposition recorded as: not known/recorded (*n* = 280), not applicable (*n* = 92), home with services (*n* = 9), home without services (*n* = 173), left against medical advice (*n* = 9), transferred to another hospital (*n* = 97), other (jail, institution care facility, mental health; *n* = 6) or if hospital discharge disposition was recorded as unknown; *n* = 1).

### Statistical analysis

Data were analyzed using the Statistical Package for the Social Sciences (SPSS, version 24). Descriptive analysis was initially performed. Categorical variables were presented using frequencies and percentages whereas the continuous variables were summarized using the mean ± standard deviation (SD), median and interquartile range (IQR).

Recoding was done for some variables due to the low frequencies of their categories. For example, the nature of injury which was originally composed of 12 categories (amputations, blood vessels, burns, crush, dislocation, fractures, internal organ, nerves, open wounds, sprains and strains, system wide and late effects, unspecified) was grouped into 7 categories with other nature of injury created by combining the following categories together: amputations, burns, crush, nerves, sprains and strains, system wide and late effects.

Depending on the cell count, Pearson’s Chi-Square or Fishers’ exact tests were used to compare the proportions of all categorical variables in terms of the outcome variable (survived: yes/no). The descriptive analysis revealed that more than 10% of the following variables (ethnicity: 11.0%, whether patient used alcohol: 13.5%, whether patient used drug: 11.1%) were categorized as being not known/not recorded. To provide accurate estimates, missing data were handled through multiple imputations. Five imputed datasets were generated. An automatic imputation method was used. This option automatically chooses an imputation method based on a scan of the data set.

A multivariate logistic regression using a forward selection procedure was conducted to find the best model that explains the association between hospital survival and all patients’ demographic and clinical characteristics. More specifically, a multivariate analysis was performed by taking into consideration all factors deemed to be clinically or statistically significant [age, gender, race, hospital teaching status, geographic region for the hospital, comorbidity, ICD-9-CM mechanism of injury E-Code, indication of the type (nature) of trauma produced by an injury, injury intentionality as defined by the CDC injury intentionality matrix, location where injury occurred, whether patient used alcohol, whether patient used drug, the patient’s primary method of payment, mode of transportation, the injury severity score reflecting the patient’s injuries directly submitted by the facility regardless of the method of calculation, ICD-9 body region as defined by the Barell injury diagnosis matrix (blood vessels, dislocation, fractures, internal organ, open wounds, unspecified, other), nature of injury as defined by the Barell injury diagnosis matrix (extremities, head and neck, spine and back, torso, unclassifiable by site), ED Thoracotomy procedure. *p* value of ≤ 0.05 was used to denote statistical significance. The c-statistic of the regression model (area under the curve = 0.934; *p* value < 0.001; 95% CI 0.924–0.943) indicated an outstanding discrimination between the two categories of the outcome (died/survived). There was no multicollinearity problem in the regression model since the variance inflation factors of all independent variables were less than 10; these ranged from 1.023 to 4.257.

## Results

**Fig. 1:**
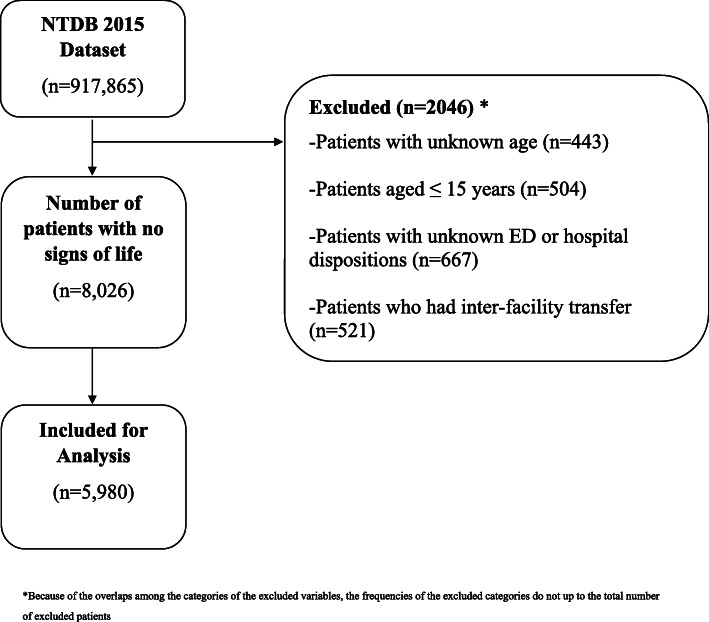
Study population

A total of 5980 patients were included in the study (Fig. 1). Only 664 patients (11.1%) survived to hospital discharge. Patients were predominantly in the age group 16–64 (84.6%) with a median age of 39 years (IQR = 26–56) (Table 1). Patients were mostly males (77.8%) and white (55.1%). Receiving hospitals were mainly University hospitals (47.7%) and community hospitals (38.9%). Most patients were admitted to Level 1 (55.5%) or Level 2 Trauma Centers (31.6%). Hospitals located in the South region of the U.S received the largest number of patients with traumatic arrests (46.8%) (Table 1). Self-pay was the most common primary method of payment (38.5%) followed by private insurances (25.3%). Most patients were transported by ground ambulance (83.9%) and most had no reported comorbidity (70.7%). Injuries were mostly blunt (56.7%) followed by penetrating (39.3%). Most injuries were unintentional (58.2%) followed by assault (29.4%). The leading mechanism of injury was MVT (38.4%) followed by Firearm (34.4%). Types of reported injuries included mainly fractures (59.0%), internal organ damage (56.2%) and open wounds (50.6%). The torso and the head and neck were the most commonly affected regions (59.4% and 52.6%, respectively). Median ISS was 19 (IQR 9–30) with more than half of the patients having severe injury (57.1%). Thoracotomy was performed on (10.1%) of patients. (Table 2).

**Table 1:** General characteristics of study population

	Frequency	%
	(*N* = 5980)	
*Age (years)*		
16–64	5062	84.6
≥ 65	918	15.4
*Gender*		
Not known/not recorded	3	0.0
Female	1327	22.2
Male	4650	77.8
*Race*		
Not known/not recorded	270	4.5
American Indian	36	0.6
Asian	96	1.6
Black or African American	1876	31.4
Native Hawaiian or Other Pacific Islander	18	0.3
White	3292	55.1
Other race	392	6.6
*Hospital teaching status*		
Community	2329	38.9
Non-teaching	801	13.4
University	2850	47.7
*Trauma designation level*		
I	3316	55.5
II	1892	31.6
III	580	9.7
IV	44	0.7
Not applicable	148	2.5
*Geographic region for the hospital*		
NORTHEAST	826	13.8
MIDWEST	1179	19.7
SOUTH	2800	46.8
WEST	1129	18.9
NA	46	0.8
*The patient’s primary method of payment*		
Not known/not recorded	402	6.7
Medicaid	728	12.2
Medicare	655	11.0
Not billed (for any reason)	61	1.0
Private/commercial insurance	1512	25.3
Self-pay	2302	38.5
Other government	119	2.0
Other	201	3.4
*Mode of transportation*		
Not known/not recorded	18	0.3
Ground ambulance	5017	83.9
Helicopter ambulance	517	8.6
Fixed-wing ambulance	6	0.1
Police	79	1.3
Public/private vehicle walk-in	278	4.6
Other	65	1.1

**Table 2:** Clinical characteristics and outcomes of patients

	Frequency	%
	(*N* = 5980)	
*Comorbidity*		
No	4228	70.7
Yes	1752	29.3
*Type (nature) of trauma produced by an injury*		
Blunt	3207	56.7
Burn	62	1.1
Other/unspecified	163	2.9
Penetrating	2226	39.3
Missing = 322 (5.4%)		
*Injury intentionality*		
Assault	1666	29.4
Self-inflicted	535	9.5
Undetermined	82	1.4
Unintentional	3292	58.2
Other	83	1.5
Missing = 322 (5.4%)		
*Mechanism of injury*		
Cut/pierce	280	4.9
Fall	687	12.1
Firearm	1946	34.4
MVT	2171	38.4
Other specified and classifiable and other specified, not elsewhere classifiable	74	1.3
Struck by, against	126	2.2
Transport, other and unspecified	171	3.0
Others	203	3.6
Missing = 322 (5.4%)		
*Alcohol use*		
No	5439	91.0
Yes	541	9.0
*Drug use*		
No	5501	92.0
Yes	479	8.0
*Nature of injury*		
Amputations	91	1.5
Blood vessels	980	16.4
Burns	90	1.5
Crush	37	0.6
Dislocation	263	4.4
Fractures	3528	59.0
Internal organ	3363	56.2
Nerves	29	0.5
Open wounds	3025	50.6
Sprains and strains	106	1.8
System wide and late effects	91	1.5
Unspecified	515	8.6
*Body region of injury*		
Extremities	2639	44.1
Head and neck	3145	52.6
Spine and back	877	14.7
Torso	3554	59.4
Unclassifiable by site	471	7.9
*Injury severity score*		
≤ 15	2438	40.8
≥ 16	3415	57.1
Not known/not recorded	127	2.1
*ED thoracotomy*		
No	5377	89.9
Yes	603	10.1
*ED disposition*		
Deceased/Expired	4491	75.1
Floor bed (general admission, non-specialty unit bed)	490	8.2
Intensive Care Unit (ICU)	366	6.1
Observation unit (24 h stays)	43	0.7
Operating room	479	8.0
Telemetry/step-down unit (less acuity than ICU)	111	1.9
*Hospital disposition*		
Not applicable	4491	75.1
Deceased/Expired	496	8.3
Discharged to home or self-care (routine discharge)	664	11.1
Transferred to other destination	319	5.3
Left against medical advice or discontinued care	10	0.2

Results of the logistic regression analysis revealed the following (Table 3): Demographic characteristics that were associated with decreased odds of survival included age group ≥ 65 (OR = 0.612, 95% CI = 0.426–0.878) and male gender (OR = 0.63, 95% CI = 0.49–0.811). Among facility related characteristics, location of hospital in South was positively associated with survival (OR = 2.055, 95% CI = 1.444–2.924) (reference Northeast). Reported comorbidity (OR = 3.201, 95% CI = 2.535–4.042), alcohol use (OR = 2.836, 95% CI = 2.053–3.917) or drug use (OR = 5.17, 95% CI = 3.695–7.234) were associated with increased survival. All injury mechanisms (with the exception of MVT) were significantly associated with increased odds of survival when compared to injury by firearm. Additionally, all methods of coverage were positively associated with survival when compared to self-pay.

**Table 3:** Factors associated with survival in traumatic arrests

	Odds ratio	95% CI	*p* value
*Age (years)* (16–64)			
≥ 65	0.612	0.426–0.878	0.008
*Gender* (Female)			
Male	0.630	0.490–0.811	< 0.001
*Geographic region for the hospital* (NORTHEAST)			
MIDWEST	1.480	0.994–2.204	0.054
SOUTH	2.055	1.444–2.924	< 0.001
WEST	1.197	0.782–1.832	0.407
*Comorbidity* (No)			
Yes	3.201	2.535–4.042	< 0.001
*Injury Intentionality as defined by the CDC InjuryIntentionality Matrix* (Assault)			
Self-inflicted	0.199	0.098–0.404	< 0.001
Unintentional	1.203	0.681–2.124	0.525
Other and undetermined	0.143	0.034–0.604	0.008
*ICD-9-CM Mechanism of Injury E-Code*			
(Firearm)	5.240	2.929–9.376	< 0.001
Cut/pierce	7.317	3.871–13.831	< 0.001
Fall	1.152	0.618–2.145	0.656
MVT^a^	2.544	0.967–6.693	0.058
Other specified and classifiable and other specified,not elsewhere classifiable	7.211	3.700–14.055	< 0.001
Struck by, against	3.082	1.432–6.634	0.004
Transport, other and unspecified others^b^	4.214	1.933–9.187	< 0.001
*Whether patient used alcohol* (No)			
Yes	2.836	2.053–3.917	< 0.001
*Whether patient used drug* (No)			
Yes	5.170	3.695 – 7.234	< 0.001
*The patient’s primary method of payment* (Self Pay)			
Medicaid	2.342	1.591–3.448	< 0.001
Medicare	2.902	1.863–4.552	< 0.001
Private/commercial insurance	3.512	2.567–4.805	< 0.001
Other government	6.973	3.466–14.029	< 0.001
Other and not billed (for any reason)	2.881	1.692–4.906	< 0.001
*Mode of transportation* (Ground Ambulance)			
Helicopter ambulance and fixed-wing ambulance	1.103	0.766–1.589	0.599
Public/private vehicle walk-in	15.649	9.077–26.978	< 0.001
Other and police	1.213	0.364–4.050	0.753
*Nature of injury as defined by the Barell Injury*			
*Diagnosis matrix* fractures (No)			
Yes	2.008	1.526–2.643	< 0.001
*Nature of injury as defined by the Barell Injury*			
*Diagnosis matrix* Internal organ (No)			
Yes	1.958	1.476–2.598	< 0.001
*Nature of injury as defined by the Barell Injury*			
*Diagnosis matrix* Unspecified (No)			
Yes	0.639	0.420–0.972	0.036
*Nature of injury as defined by the Barell Injury*			
*Diagnosis matrix* Other (No)			
Yes	2.336	1.506–3.624	< 0.001
*ICD-9 body region as defined by the Barell*			
*Injury diagnosis matrix* Extremities (No)			
Yes	1.471	1.152–1.879	0.002
*ICD-9 body region as defined by the Barell*			
*Injury diagnosis matrix* Head and Neck (No)			
Yes	0.405	0.314–0.523	< 0.001
*ICD-9 body region as defined by the Barell*			
*Injury diagnosis matrix* Spine and Back (No)			
Yes	1.652	1.205–2.264	0.002
*ICD-9 body region as defined by the Barell*			
*Injury diagnosis matrix* Torso (No)			
Yes	0.473	0.356–0.629	< 0.001
*ICD-9 body region as defined by the Barell*			
*Injury diagnosis matrix* Unclassifiable by site (No)			
Yes	0.273	0.154–0.483	< 0.001
*The Injury Severity Score reflecting the patient’sinjuries directly submitted by the facility regardlessof the method of calculation* (≤ 15)			
≥ 16	0.092	0.068–0.125	< 0.001
*ED thoracotomy* (No)			
Yes	0.334	0.169–0.662	0.002

Variables that were included in the model are: age, gender, race, hospital teaching status, Geographic region for the hospital, comorbidity, ICD-9-CM Mechanism of Injury E-Code, Indication of the type (nature) of trauma produced by an injury, Injury Intentionality as defined by the CDC Injury Intentionality Matrix, Location where injury occurred, Whether patient used alcohol, Whether patient used drug, the patient’s primary method of payment, Mode of Transportation, The Injury Severity Score reflecting the patient’s injuries directly submitted by the facility regardless of the method of calculation, ICD-9 body region as defined by the Barell Injury Diagnosis Matrix (Blood vessels, Dislocation, Fractures, Internal organ, Open wounds, Unspecified, Other), Nature of injury as defined by the Barell Injury Diagnosis Matrix (Extremities, Head and Neck, Spine and Back, Torso, Unclassifiable by site), ED Thoracotomy

^a^MVT is the combination of the following variables: MVT Motorcyclist and MVT Occupant and MVT Other and MVT Pedal cyclist and MVT Pedestrian and MVT Unspecified

^b^Others is the combination of the following variables: Drowning/submersion and Fire/flame and Hot object/substance and Machinery and Pedal cyclist, other and Pedestrian, other and Natural/environmental, Bites and stings and Natural/environmental, Other and Overexertion and Poisoning and Suffocation

Injury related factors associated with survival were also identified. Self-inflicted and other or undetermined types of injuries were associated with decreased survival when compared to assault type of injuries. Presence of specific types of injuries such as fractures (OR = 2.008, 95% CI = 1.526–2.643), internal organ damage (OR = 1.958, 95% CI = 1.476–2.598), and other nature of injury—a variable that was created by combining some injuries together—(OR = 2.336, 95% CI = 1.506–3.624) were all associated with higher odds of survival. Patients who had unspecified (OR = 0.639, 95% CI = 0.42–0.972) injuries were less likely to survive. Patients who had injuries in their extremities (OR = 1.471, 95% CI = 1.152–1.879) or spine and back (OR = 1.652, 95% CI = 1.205–2.264) were more likely to survive, whereas those who had injuries in their head and neck (OR = 0.405, 95% CI = 0.314–0.523) or torso (OR = 0.473, 95% CI = 0.356–0.629), or in unclassifiable site (OR = 0.273, 95% CI = 0.154–0.483) had poor outcome. Injury severity score ≥ 16 was associated with decreased odds of survival from traumatic arrest (OR = 0.092, 95% CI = 0.068–0.125). Finally, ED thoracotomy was as well associated with a lower odds of survival (OR = 0.334, 95% CI = 0.169–0.662).

## Discussion

Research involving traumatic arrests is rare. This study examined outcomes of patients with traumatic arrests treated in US trauma centers and identified factors associated with survival in this population using the largest trauma database in the US. These factors have not been previously described in the literature.

The overall survival to hospital discharge among patients with traumatic arrests was 11.1%. This rate is slightly lower than the rate of 12.5% reported by Ahmed et al. [5] but higher than other survival rates reported in earlier studies [13, 18]. Young, white and male individuals continue to be mostly affected by traumatic arrests [1, 5, 11]. Improved survival in traumatic arrests presenting to U.S hospitals has been previously attributed to the increasing frequency of emergency interventions such as ED thoracotomy and other procedures [19]. Variation in survival rates of patients with traumatic arrest is however mostly related to differences in study sample selections with most studies including in the denominator arrests that are not transported to hospitals [20]. Our study included only patients who were transported to a trauma center which might have overestimated the survival rate since patients declared dead on scene are not usually included in the NTDB registry.

There were several factors associated with increased survival in patients with traumatic arrests. Demographic factors positively associated with survival included age group (16–64) (compared to age ≥ 65) and female gender. This differs from previous studies [5, 18, 21] where no similar associations were reported. Patients in the younger age are usually expected to have better odds of survival because of lower comorbidities. Female gender was significantly associated with survival in this population. A previous study did not identify significant association between female gender and outcomes in severely injured patients [22]. Age stratification was however done in that study to account for hormonal status. This finding in our study needs further examination with the potential role of other unmeasured confounders such as role of hormones based on age category (pre vs post-menopausal) and obesity etc.

Several injuries related characteristics that are relevant to treating physicians were also significantly associated with survival.

Type of trauma (blunt vs penetrating), which is mainly used for classification in trauma studies, was not significantly associated with survival similar to other previous studies [5, 18, 23]. This is in contrast with other studies that reported better outcomes with either penetrating [24] or with blunt injuries [25]. The sample used for our study was however heterogeneous and not restricted to a specific type which might explain this difference.

Additional classification by mechanisms of injuries revealed that most mechanisms were also positively associated with survival when compared to injury by firearm. While fall-related injuries were previously shown to be associated with increased survival when compared to MVT in blunt traumatic arrests [24, 26] this study is the first to examine all available mechanisms in traumatic arrests. The case fatality rate of firearm is usually much higher than any other mechanism of injury [27, 28] and is related to several factors including number of entrance wounds, range and site of entrance wounds and intentionality [22, 29]. The study findings highlight the need for better understanding of firearm related injuries and for developing preventive measures targeted to improve survival in this population.

Injury body region was also found to be significantly associated with survival: Injuries to vital locations such as head and neck, or torso were associated with poor outcomes. This was expected because of the risk of bleeding and hemorrhagic shock from damage to vital organs [30] and is in line with the ATLS approach to management of trauma patients by prioritizing management according to life-threatening injuries [31]. Other factors were also found to be positively associated with survival such as specific types of injuries (fractures, internal organ damage) in addition to alcohol or drug use. These are more likely related to reporting of such data elements in patients who survive after the initial resuscitation measures. Such patients are expected to have a more detailed documentation of minor injuries or better description of other elements contributing to the injury event.

As expected an injury severity score (ISS < 16) was associated with higher survival in traumatic arrest patients. This finding is in line with previous studies [5, 13, 18] and validates the need to incorporate ISS in outcome predictive models in not only trauma patients but also in traumatic arrests to avoid futility of extreme measures in resuscitation. ED thoracotomy was negatively associated with survival in our patient population. This is expected since the procedure is highly time dependent and considered as a last resort [19]. Thus, if not used in the appropriate population i.e. penetrating chest injuries, ED thoracotomy may be ineffective [9], as such its success rate misrepresented, especially in a heterogeneous population such as ours.

Our study also identified that hospital location in the South was associated with increased survival (reference Northeast) for patients with traumatic arrests. While improved outcomes in trauma patients have been previously linked to geographic clustering of trauma centers which are primarily located in the Northern area [32] and where patients benefit from the greatest access to trauma Level I and II centers within 45 and 60 min [33], our study did not identify such association. In our study sample, more hospitals were located in the South (47.2%) than in the Northeast (13.9%). Patients in the South were more likely to survive as compared to those in the Northeast (17.1% vs. 13.4%) and additional stratification by mortality status revealed that disproportionate distribution of the participant hospitals may be responsible for this apparent survival differences between the two regions (Only two patients with firearm injury survived to hospital discharge in the Northeast as compared to 41 patients in the South). Further research should examine closely the impact of hospital region on survival in trauma patients.

Financial coverage status was also significantly associated with survival. When compared to self-payment or uninsured, all other methods of coverage were positively associated with survival. The available literature reports contradicting findings on financial coverage and association with survival in trauma patients. Greene et al. concluded that insurance status was a predictor of outcome with uninsured patients being at higher risk of death in both blunt and penetrating trauma [34] while Lober et al. [21] noted better survival in patients with no insurance coverage attributing this finding to potentially larger proportion of healthy patients in the uninsured group. In this study of traumatic arrests any insurance status (compared to uninsured) was associated with improved outcomes. Further research is needed to clarify the reasons for this disparity such as examining resources utilization including but not limited to access to surgical procedures.

This study has potential limitations related to its retrospective nature and to availability of data reported in the database. The NTDB uses ICD-9 coding for the data retrieved from different hospitals and is like other national databases subject to coding variations and errors. This study did not include traumatic arrests that were not transported to a trauma center which might have led to overestimation of the survival rate in this group of patients. The NTDB also uses “convenience samples” from disproportionate number of large to small hospitals that contribute to the database. This unequal sample size across regions and the lack of weighting should be taken into considerations when comparing outcomes across different US regions. Another limitation is related to absence of NTDB of important clinical factors (no-flow time, low-flow time) which have been shown to be important predictors of survival. Adding these data elements to trauma registries can improve future trauma studies examining outcomes.

Despite these limitations, the NTDB is the largest database for trauma in the United States of America collecting data from over 900 hospitals and the study findings can be generalized to hospitals in the US and to other settings with similar trauma systems. This study also is comprehensive in examining a heterogeneous sample of traumatic arrests that is reflective of Traumatic arrests treated in different trauma centers in the US. The findings of this study are also important for physicians managing trauma patients in the US and in other areas of the world and familiarity with the identified predictors can allow for better assessment of prognosis when treating traumatic arrests.

## Conclusion

Patients with traumatic arrests continue to have poor outcomes with only 11.1% surviving to hospital discharge. Several factors were identified to be positively associated with survival in this population. Survival is higher for younger age group, female gender and with any type of insurance coverage. Patients with traumatic arrests from firearm mechanisms experience poor survival. These findings are important for future studies examining closely different predictors, and for devising injury prevention strategies. More evidence is needed to guide trauma management protocols and initiation of resuscitation to improve outcomes further in traumatic arrests.

## Data Availability

The datasets generated and/or analyzed during the current study are available in the National Trauma Data Bank (collected in 2015 and released in 2017) repository: https://www.facs.org/quality-programs/trauma/tqp/center-programs/ntdb.
